# Mental Health–Related Emergency Department Visits Among Children Aged <18 Years During the COVID-19 Pandemic — United States, January 1–October 17, 2020

**DOI:** 10.15585/mmwr.mm6945a3

**Published:** 2020-11-13

**Authors:** Rebecca T. Leeb, Rebecca H. Bitsko, Lakshmi Radhakrishnan, Pedro Martinez, Rashid Njai, Kristin M. Holland

**Affiliations:** ^1^Division of Human Development and Disability, National Center on Birth Defects and Developmental Disabilities, CDC; ^2^Innovation, Technology, and Analytics Task Force, CDC COVID-19 Response Team; ^3^Division of Injury Prevention, National Center for Injury Prevention and Control, CDC; ^4^Community Interventions and Critical Populations Task Force, CDC COVID-19 Response Team; ^5^Division of Overdose Prevention, National Center for Injury Prevention and Control, CDC.

Published reports suggest that the coronavirus disease 2019 (COVID-19) pandemic has had a negative effect on children’s mental health (*1*,*2*). Emergency departments (EDs) are often the first point of care for children experiencing mental health emergencies, particularly when other services are inaccessible or unavailable (*3*). During March 29–April 25, 2020, when widespread shelter-in-place orders were in effect, ED visits for persons of all ages declined 42% compared with the same period in 2019; during this time, ED visits for injury and non-COVID-19–related diagnoses decreased, while ED visits for psychosocial factors increased (*4*). To assess changes in mental health–related ED visits among U.S. children aged <18 years, data from CDC’s National Syndromic Surveillance Program (NSSP) from January 1 through October 17, 2020, were compared with those collected during the same period in 2019. During weeks 1–11 (January 1–March 15, 2020), the average reported number of children’s mental health–related ED visits overall was higher in 2020 than in 2019, whereas the proportion of children’s mental health–related visits was similar. Beginning in week 12 (March 16) the number of mental health–related ED visits among children decreased 43% concurrent with the widespread implementation of COVID-19 mitigation measures; simultaneously, the proportion of mental health–related ED visits increased sharply beginning in mid-March 2020 (week 12) and continued into October (week 42) with increases of 24% among children aged 5–11 years and 31% among adolescents aged 12–17 years, compared with the same period in 2019. The increased proportion of children’s mental health–related ED visits during March–October 2020 might be artefactually inflated as a consequence of the substantial decrease in overall ED visits during the same period and variation in the number of EDs reporting to NSSP. However, these findings provide initial insight into children’s mental health in the context of the COVID-19 pandemic and highlight the importance of continued monitoring of children’s mental health throughout the pandemic, ensuring access to care during public health crises, and improving healthy coping strategies and resiliency among children and families.

CDC analyzed NSSP ED visit data, which include a subset of hospitals in 47 states representing approximately 73% of U.S. ED visits.* Mental health–related ED visits among children aged <18 years was a composite variable derived from the mental health syndrome query of the NSSP data for conditions likely to result in ED visits during and after disaster events (e.g., stress, anxiety, acute posttraumatic stress disorder, and panic).^†^ Weekly numbers of mental health–related ED visits and proportions of mental health–related ED visits (per 100,000 pediatric ED visits^§^) were computed overall, stratified by age group (0–4, 5–11, and 12–17 years) and sex, and compared descriptively with the corresponding weekly numbers and proportions for 2019. Numbers and proportions of visits were compared during calendar weeks 1–11 (January 1–March 14, 2020) and weeks 12–42 (March 15–October 17, 2020) (before and after a distinct decrease in overall ED visits reported beginning in week 12 in 2020)^¶^ (*4*). Analyses are descriptive and statistical comparisons were not performed.

The number of children’s mental health–related ED visits decreased sharply from mid-March 2020 (week 12, March 15–21) through early April (week 15, April 5–11) and then increased steadily through October 2020. (Figure 1). During the same time, the overall proportion of reported children’s ED visits for mental health–related concerns increased and remained higher through the end of the reporting period in 2020 than that in 2019 (Figure 1). The proportion of mental health–related ED visits among children increased 66%, from 1,094 per 100,000 during April 14–21, 2019 to 1,820 per 100,000 during April 12–18, 2020 (Supplementary Figure 1, https://stacks.cdc.gov/view/cdc/96609). Although the average reported number of children’s mental health–related ED visits overall was 25% higher during weeks 1–11 in 2020 (342,740) than during the corresponding period in 2019 (274,736), the proportion of children’s mental health–related visits during the same time was similar (1,162 per 100,000 in 2020 versus 1,044 per 100,000 in 2019). (Table). During weeks 12–42, 2020 (mid-March–October) however, average weekly reported numbers of total ED visits by children were 43% lower (149,055), compared with those during 2019 (262,714), whereas the average proportion of children’s mental health–related ED visits was approximately 44% higher in 2020 (1,673 per 100,000) than that in 2019 (1,161 per 100,000).

**FIGURE 1:**
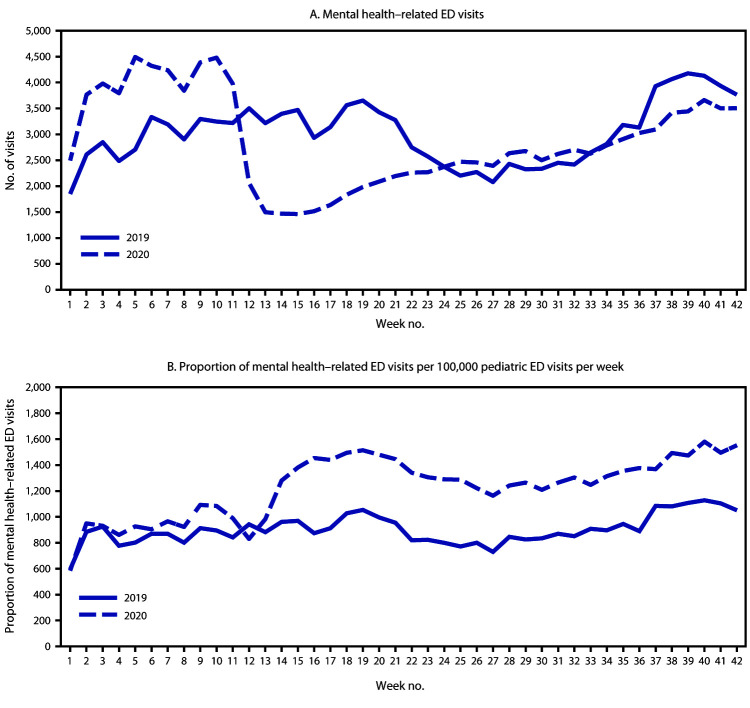
Weekly number of emergency department (ED) mental health–related visits (A) and proportion of (B) children’s mental health–related ED visits per total ED visits* among children aged <18 years — National Syndromic Surveillance Program, United States, January–October 2019 and 2020 * Proportion of mental health–related ED visits = number of ED visits for children’s mental health/total number of pediatric ED visits x 100,000.

**TABLE:** Average number and proportions* of emergency department (ED) visits and mental health–related ED visits^†^ among children aged <18 years — National Syndromic Surveillance Program (NSSP), United States, 2019–2020

Surveillance period/indicators	2019				2020			
	Age group, yrs				Age group, yrs			
	All <18	0–4	5–11	12–17	All <18	0–4	5–11	12–17
**Weeks 1–42** ^§^								
Average weekly total ED visits	265,863	110,002	81,133	74,728	199,782	78,742	59,660	61,380
Average weekly mental health–related ED visits	3,025	80	625	2,320	2,872	54	522	2,296
Mental health–related ED visits per 100,000 visits	1,130	73	762	3,084	1,539	75	919	3,863
**Weeks 1–11** ^¶^								
Average weekly total ED visits	274,736	118,926	83,924	71,886	342,740	143,789	107,049	91,902
Average weekly mental health–related ED visits	2,876	82	594	2,200	3,974	80	821	3,073
Mental health–related ED visits per 100,000 visits	1,044	69	707	30,45	1,162	56	769	3,333
Weeks 12–42**								
Average weekly total ED visits	262,714	106,835	80,143	75,736	149,055	55,661	42,844	50,550
Average weekly mental health–related ED visits	3,078	79	635	2,363	2,481	45	416	2,020
Mental health–related ED visits per 100,000 visits	1,161	75	782	3,098	1,673	81	972	4,051

* Average proportion of ED visits for children’s mental health = (average number of ED visits for children’s mental health/average total number of ED visits for the same age or sex population [e.g., children aged 18 years]) x 100,000. All numbers have been rounded to the nearest whole number.

^†^ Mental health–related ED visits were defined using NSSP’s Syndrome Definition (SD) Subcommittee community-developed syndrome definition for mental health conditions likely to increase in ED frequency during and after natural or human-caused disaster events. This syndrome definition attempts to leverage only mental health conditions and presentations that showed increases in visit frequency after select disasters in the United States. There are no disaster-related terms inherent to this query. The query has been added to NSSP BioSense Platform Electronic Surveillance System for the Early Notification of Community-based Epidemics as a Chief Complaint and Discharge Diagnosis category. https://knowledgerepository.syndromicsurveillance.org/disaster-related-mental-health-v1-syndrome-definition-subcommittee.

^§^ Weeks 1–42 in 2019 correspond to December 30, 2018–October 19, 2019; weeks 1–42 in 2020 correspond to December 29, 2019–October 17, 2020.

^¶^ Weeks 1–11 in 2019 correspond to December 30, 2018–March 16, 2019; weeks 1–11 in 2020 correspond to December 29, 2019–March 14, 2020.

** Weeks 12–42 in 2019 correspond to March 17–October 19, 2019; weeks 12–42 in 2020 correspond to March 15–October 17, 2020.

Adolescents aged 12–17 years accounted for the largest proportion of children’s mental health–related ED visits during 2019 and 2020 (Figure 2). During weeks 12–42, 2020, the proportion of mental health–related visits for children aged 5–11 years and adolescents aged 12–17 years increased approximately 24% and 31%, respectively compared with those in 2019; the proportion of mental health–related visits for children aged 0–4 years remained similar in 2020. (Table.) The highest weekly proportion of mental health–related ED visits occurred during October for children aged 5–11 years (week 42; 1,177 per 100,000) and during April (week 16) for adolescents aged 12–17 years (4,758 per 100,000) (Figure 2).

**FIGURE 2:**
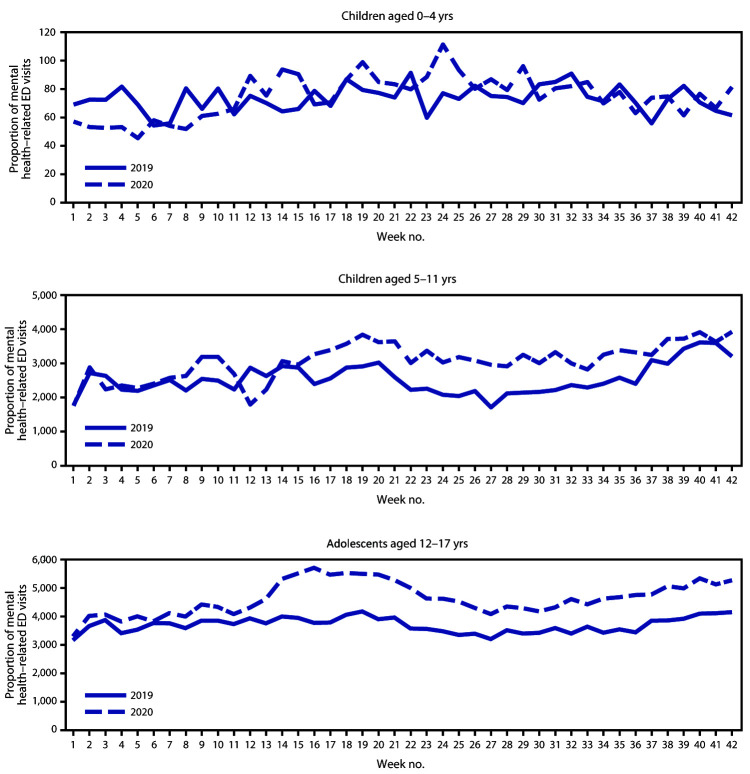
Weekly proportion of mental health–related emergency department (ED) visits* per total ED visits among children aged <18 years, by age group — National Syndromic Surveillance Program, United States, January–October 2019 and 2020 * Proportion of mental health–related ED visits = number of ED visits for children’s mental health/total number of pediatric ED visits x 100,000.

During 2019 and 2020, the proportion of mental health–related ED visits was higher among females aged <18 years than it was among males (Supplementary Figure 2, https://stacks.cdc.gov/view/cdc/96610). Similar patterns of increasing proportions of mental health–related ED visits were observed in 2020 for males and females, with increases beginning mid-March and continuing through October.

## Discussion

Substantial declines in the overall reported numbers of children’s mental health–related ED visits occurred in 2020 during mid-March to early May, coincident with the widespread implementation of community mitigation measures** enacted to prevent COVID-19 transmission (e.g., school closures and restrictions to nonemergent care) and decreases in overall ED visits for the same period (*4*). A previous report found the mean weekly number of ED visits for children aged <14 years declined approximately 70% during March 29–April 25, 2020, relative to the corresponding period in 2019 (*4*). Further, the mean number of weekly ED visits for persons of all ages decreased significantly for asthma (–10%), otitis media (–65%), and sprain- and strain-related injuries (–39%), and mean weekly ED visits for psychosocial factors increased 69% (*4*). This report demonstrates that, whereas the overall number of children’s mental health–related ED visits decreased, the proportion of all ED visits for children’s mental health–related concerns increased, reaching levels substantially higher beginning in late-March to October 2020 than those during the same period during 2019. Describing both the number and the proportion of mental health–related ED visits provides crucial context for these findings and suggests that children’s mental health warranted sufficient concern to visit EDs during a time when nonemergent ED visits were discouraged.

Many children receive mental health services through clinical and community agencies, including schools (*5*). The increase in the proportion of ED visits for children’s mental health concerns might reflect increased pandemic-related stress and unintended consequences of mitigation measures, which reduced or modified access to children’s mental health services (*2*), and could result in increased reliance on ED services for both routine and crisis treatment (*3*). However, the magnitude of the increase should be interpreted carefully because it might also reflect the large decrease in the number and proportion of other types of ED visits (e.g., asthma, otitis media, and musculoskeletal injuries) (*4*) and variation in the number of EDs reporting to NSSP.

Adolescents aged 12–17 years accounted for the highest proportion of mental health–related ED visits in both 2019 and 2020, followed by children aged 5–11 years. Many mental disorders commence in childhood, and mental health concerns in these age groups might be exacerbated by stress related to the pandemic and abrupt disruptions to daily life associated with mitigation efforts, including anxiety about illness, social isolation, and interrupted connectedness to school (*5*). The majority of EDs lack adequate capacity to treat pediatric mental health concerns (*6*), potentially increasing demand on systems already stressed by the COVID-19 pandemic. These findings demonstrate continued need for mental health care for children during the pandemic and highlight the importance of expanding mental health services, such as telemental health and technology-based solutions (e.g., mobile mental health applications) (*5*,*7*).

The findings in this report are subject to at least three limitations. First, the proportions presented should be interpreted with caution because of variations affecting the denominators used to calculate proportions. Children’s mental health–related ED visits constitute a small percentage of all pediatric ED visits (1.1% in 2019 and 1.4% in 2020), increasing susceptibility of rates to decreases in ED visits during the pandemic. In addition, NSSP ED participation can vary over time; however, analyzing number of visits and proportion of total ED visits provides context for observed variation. Second, NSSP data are not nationally representative; these findings might not be generalizable beyond those EDs participating in NSSP. Further, usable information on race and ethnicity was not available in the NSSP data. Finally, these data are subject to under- and overestimation. Variation in reporting and coding practices can influence the number and proportion of mental health–related visits observed. ED visits represent unique events, not individual persons, and as such, might reflect multiple visits for one person. The definition of mental health focuses on symptoms and conditions (e.g., stress, anxiety) that might increase after a disaster in the United States and might not reflect all mental health–related ED visits. Still, these data likely underestimate the actual number of mental health–related health care visits because many mental health visits occur outside of EDs.

Children’s mental health during public health emergencies can have both short- and long-term consequences to their overall health and well-being (*8*). This report provides timely surveillance data concerning children’s mental health in the context of the COVID-19 pandemic. Ongoing collection of a broad range of children’s mental health data outside the ED is needed to monitor the impact of COVID-19 and the effects of public health emergencies on children’s mental health. Ensuring availability of and access to developmentally appropriate mental health services for children outside the in-person ED setting will be important as communities adjust mitigation strategies (*3*). Implementation of technology-based, remote mental health services and prevention activities to enhance healthy coping and resilience in children might effectively support their well-being throughout response and recovery periods (*5*,*7*). CDC supports efforts to promote the emotional well-being of children and families and provides developmentally appropriate resources for families to reduce stressors that might contribute to children’s mental health–related ED visits^††^ (*9*).

**Summary**

**What is already known about this topic?**

Emergency departments (EDs) are often the first point of care for children’s mental health emergencies. U.S. ED visits for persons of all ages declined during the early COVID-19 pandemic (March–April 2020).

**What is added by this report?**

Beginning in April 2020, the proportion of children’s mental health–related ED visits among all pediatric ED visits increased and remained elevated through October. Compared with 2019, the proportion of mental health–related visits for children aged 5–11 and 12–17 years increased approximately 24%. and 31%, respectively.

**What are the implications for public health practice?**

Monitoring indicators of children's mental health, promoting coping and resilience, and expanding access to services to support children's mental health are critical during the COVID-19 pandemic.

## Footnotes

* The National Syndromic Surveillance Program (NSSP) is a network developed and maintained by CDC, state and local health departments, and academic and private sector health partners to collect electronic health data in real time. NSSP includes ED visit data from a subset of hospitals in 47 states (all but Hawaii, South Dakota, and Wyoming). https://www.cdc.gov/nssp/participation-coverage-map.html; https://www.cdc.gov/nssp/calculations-for-coverage.html.

† Mental health–related ED visits were defined using the NSSP Syndrome Definition (SD) Subcommittee community-developed syndrome definition for mental health conditions likely to increase in emergency department frequency during and after natural or human-caused disaster events. This syndrome definition attempts to leverage only mental health conditions and presentations that showed increases in visit frequency after select disasters in the United States. There are no disaster-related terms inherent to this query. The query has been added to NSSP BioSense Platform Electronic Surveillance System for the Early Notification of Community-based Epidemics as a Chief Complaint and Discharge Diagnosis category. https://knowledgerepository.syndromicsurveillance.org/disaster-related-mental-health-v1-syndrome-definition-subcommittee.

§ Average proportion of ED visits for children’s mental health = (average number of ED visits for children’s mental health/average total number of ED visits for the same age or sex population [e.g., children aged <18 years]) x 100,000.

¶ To decrease the effect of differential reporting, this analysis was restricted to only include hospitals sending diagnosis codes at patient discharge that are >75% complete and informative, with <20% standard deviation in their values over the previous 2 years.

** https://www.whitehouse.gov/wp-content/uploads/2020/03/03.16.20_coronavirus-guidance_8.5x11_315PM.pdf; https://www.cdc.gov/coronavirus/2019-ncov/community/community-mitigation.html.

††
https://www.cdc.gov/coronavirus/2019-ncov/daily-life-coping/parental-resource-kit/ .
